# Cryopreservation Techniques for Ram Sperm

**DOI:** 10.1155/2022/7378379

**Published:** 2022-04-30

**Authors:** Amit Saha, Mohammad Asaduzzaman, Farida Yeasmin Bari

**Affiliations:** ^1^Department of Surgery and Obstetrics, Bangladesh Agricultural University, Mymensingh 2202, Bangladesh; ^2^Department of Livestock Services, Farmgate, Dhaka 1215, Dhaka, Bangladesh

## Abstract

Germplasm storage and transportation in artificial insemination (AI) and other advanced technologies are facilitated by cryopreservation. In reproduction, the cryopreservation of sperm allows it to be transported across vast distances and used even after the sire's death. However, the technique of cryopreservation might damage sperm and limit their activity. Several cryobiological investigations have reported that the integrity of the sperm membrane is frequently involved in the physical and biological elements that affect sperm survival at low temperatures during the cryopreservation process. However, successful cryopreservation of ram sperm is still a work in progress because a considerable percentage of sperm do not survive the freezing and thawing process. Sperms are destroyed during cryopreservation of semen due to varying concentrations of cryoprotective chemicals and if semen is not cooled at optimal cooling rates. Hence, it is crucial to know the optimum cooling rates with freezing and thawing protocols for maximum recovery of viable and functional sperm cells for a successful cryo-freezing of ram spermatozoa. Therefore, the current study compiled and compared the research on the impact of different cryopreservation procedures, cooling rates, equilibration time, and thawing protocols on post-thaw ram semen quality.

## 1. Introduction

Cryopreservation of sperm is one of the most important tools in animal research for improving reproductive technology [1]. Cryopreservation of mammalian sperm is a complicated technique that requires a proper balance of many factors to achieve optimal outcomes. The ram spermatozoa contain a low intramembrane cholesterol-to-phospholipid ratio compared to other species. Henceforth, cold-shock sensitivity in ram spermatozoa is higher than in other species [2]. Diluents, dilution-cooling-freezing, and thawing techniques all play a role in the success of ram semen cryopreservation [3, 4]. Moreover, the need to improve the reproductive efficacy of breeding with cryopreserved semen could involve using better freezing methods to improve post-thaw sperm quality. Sperm cells undergo biochemical and functional changes due to long-term spermatozoa storage, limiting their ability to fertilize [5]. The acrosome, nucleus, mitochondria, axoneme, and plasma membrane are also affected by rapid temperature changes, such as cold shock and the creation and dissolution of ice during the freezing-thawing process [6, 7]. To prevent intracellular crystallization, semen is routinely diluted with a cryoprotectant extender.

Egg yolk is employed in non-penetrating cryoprotectants that successfully protect sperm function (motility, viability, and acrosome integrity) after thawing, while glycerol is the most commonly used penetrating cryoprotectant [8–11]. In glycerol, the concentration that provides the best post-thaw survival rate is between 3% and 7%, while in egg yolk, it is between 5 and 20%. [12–14]. Sperm cryo survival is also influenced by freezing and thawing rates [15, 16]. To minimize cryoinjuries such as disruption of the sperm plasma membrane and DNA structure caused by considerable intracellular ice formation, as well as changes in intracellular pH and ionic composition, an adequate cooling rate must be identified [15, 17, 18].

The rapid cooling of sperm from 30 to 40 degrees Celsius causes “cold shock” damage [19]. Temperature variations during cooling produce stress on sperm membranes, causing phase shifts in lipids and a change in the functional condition of sperm membranes. Temperatures between 5° and −15°C are known to cause a significant phase change [20], and this could be the perfect temperature range for temperature-dependent damage. The appropriate freeze rate to minimize intracellular ice crystal formation is slow enough to allow water to leave the cells yet fast enough to prevent severe cell dehydration and the solution effect [21]. For the first time, Polge [22] claimed that most spermatozoa destruction occurs in a crucial temperature zone between −15 and −30°C and that if cooling rates are not adequate, all cells may be killed by −80°C. The phase of supercooling (0°C to −5°C) and the development of ice crystals (−6°C to −15°C) are the two main temperature ranges where sperm are damaged during freezing [23]. Mazur [24] also believes that during freezing and thawing, damage to sperm membranes occurs in the temperature range of −15°C to −60°C, which is known as the crucial temperature range.

Standard cryopreservation techniques involve cooling spermatozoa contained in cryoprotective media in plastic straws in the vapor phase over liquid nitrogen. Different protocols have accurate data on freezing ram semen using programmable biofreezers, liquid nitrogen vapor, and dry ice. The techniques of collection of semen, category of semen extender, whether or not the seminal plasma should be separated before freezing, how lengthy the semen should be equilibrated before freezing (2 h or 4 h), the ultimate sperm concentration (ranging from 50 × 10^6^/mL to 1000 × 10^6^/mL), straw size (0.25 mL, 0.5 mL), and freezing methods (dry ice for pellets, vapor freezing or controlled freezing for the semen straws). All of these variables could impact the quality of sperm after they have been thawed. Because of these and other differences, this study aims to look at seminal plasma effects, semen cryopreservation media, cryoprotectants, semen dilution, cooling rate, and thawing rate, as well as the effect of the straw freezing method (vapor freezing, programmable freezing, or dry ice) on post-thaw ram semen quality.

## 2. Semen Collection

Semen collection aims to get the maximum sperm cells with the best quality possible from each ejaculate. Semen is collected mainly through artificial vaginal (AV) or electroejaculation (EE) in small ruminants. Semen collection by AV is the preferred method for sheep [25, 26]. The AV method of semen collection is more appropriate and simple to use and produces ejaculate with sperm quality comparable to natural ejaculate [27, 28]. Warm water in the AV provides thermal stimulation, while pressure in the AV stimulates the glans penis mechanically [27]. The main disadvantage of this procedure is that it necessitates a ram with good libido, the ability to mount females or treasure, and prior ejaculation training in AV [28]. As an alternative to AV, the EE method can be used [29]. An electrode probe (6–12V) is used to apply 3–5 bouts of short stimulation (3–8 sec) at 15–20 sec intervals to the nerve of the accessory glands of the reproductive organ in the EE method [30].

The EE method eliminates the need for ram training, boosts ejaculates quickly, and, most crucially, collects sperm from superior males who are unable to mount due to injury or old age [31]. The main disadvantage of this procedure is that it is stressful for the animal, which is a big problem in animal welfare [31, 32]. The qualities of sperm differ depending on the method of collecting; nevertheless, when AV is used, the results are more favorable [33]. The AV method of semen collection in rams yielded somewhat higher semen volume, sperm concentration, total sperm number, percentage of normal sperm, and wave motion than the EE method [32, 34]. The method of semen collection has an impact on the composition of seminal plasma and the volume and features of the sperm [32]. AV-collected spermatozoa in sheep were more resistant to cold shock than EE-collected spermatozoa [32]. Furthermore, it has been reported that seminal plasma proteins play an essential role in preventing cold shocks without harming the sperm cell membrane [35]. However, semen composition may change during the EE procedure, affecting sperm sample cryo resistance.

## 3. Sperm Dilution and Concentration

In order to achieve a high fertility rate with the fewest number of inseminations and sperm per insemination, a semen sample must be appropriately diluted to guarantee that sufficient amounts of sperm and diluent are present to accommodate the cells in an insemination straw. It was common practice to dilute farm animal sperm samples with precise amounts of diluents or to dilute them to a specified spermatozoa concentration. It has been effectively utilized dilution rates of 1 : 1–1 : 23 (v/v; semen to diluent) [30, 36, 37]. For comparison, sperm concentration might be an ideal way to dilute semen. There have been instances of sperm being properly frozen and adequate fertility with samples ranging from 80 to 500 × 10^6^ cells/ml [36–39].

## 4. Sperm Cryopreservation

Sperm cryopreservation is an optional approach for keeping spermatozoa from the best donor for unlimited periods, allowing for the preservation of genetic pools and the most efficient use of sperm dosages using AI. The principle of sperm cryopreservation is to halt the cellular metabolic rate of sperm cells frozen in LN2 (−196°C) and then thaw the sperm to restore their functional survival [40]. The entire procedure necessitates a high level of adaptation to several functions including dilution, incubation, cooling, freezing, and thawing [26, 41, 42]. The spermatozoa undergo varying degrees of ultrastructural, biochemical, and functional damage, resulting in decreased sperm motility, membrane integrity, and fertilizing ability [19, 43]. The production of extracellular and intracellular ice in the sperm cell is thought to cause these damages. The unfrozen solution induces efflux of intracellular water through ice crystallization, cell contraction, and possibly an influx of ions. The plasma, acrosome membranes, mitochondrial sheath, and axoneme of the sperm cell are more damaged in frozen-thawed semen [10, 19, 32].

The biochemical properties of sperm are also affected by the cryopreservation technique. There is a release of glutamic-oxaloacetic transaminase (GOT), inactivation of hyaluronidase, acrosin enzyme, and an increase in sodium. There are decreases in phosphatase activity, acrosomal proteolytic activity, cholesterol protein, potassium content, and adenosine triphosphate (ATP) and adenosine diphosphate (ADP) synthesis, as well as losses of amino acids and prostaglandins [2]. The total effect of cryopreservation on sperm cells is influenced by both internal and extrinsic variables. The inherent sperm cell properties, such as the cell geometry (diameter, volume, relation surface volume), sperm hydration condition, plasma membrane permeability to water and cryoprotectants, and spermatozoa age or maturation state, are all intrinsic variables [44, 45]. The cholesterol/phospholipids ratio, the number of lipids in the bilayer, the degree of hydrocarbon chain saturation, and the protein/phospholipid ratio are essential considerations [42]. In this context, sperm from bulls, rams, and boars has a greater unsaturated to saturated fatty acid ratio and is more susceptible.

In contrast, sperm from rabbits, dogs, and humans has a lower ratio and is more resistant. Cooling and freezing rates, cryoprotective agent type and concentration, extender composition, dilution rate, the temperature at which glycerol is added to the semen, equilibration duration, and thawing rate are all extrinsic factors [2, 45–47]. The osmotic tolerance limitations of ram sperm are similar to those of boar and bull sperm [48]. Ram sperm cells are more sensitive to extreme temperature changes during the freezing process and suffer more damage than bull sperm cells [10, 32, 49]. The cryopreservation of ram sperm is not a simple process. The motility of rams' spermatozoa is better retained after slow and quick freezing than the morphological integrity of spermatozoa. The mitochondrial architecture is affected by freezing and thawing. However, the tail filament and fibrils do not display any changes [10].

## 5. Cryopreservation Diluents

Diluents are the media used to increase ejaculate volume and retain sperm fertility for the longest time possible, allowing multiple female animals to be inseminated over a long period [10, 50]. The semen diluent should be able to offer nutrients as an energy source, protect cells from temperature-related damage, provide a buffer to prevent damaging pH shifts [51], suppress bacterial development, and protect sperm cells during preservation [50, 52]. The diluent should have maximum solubility in water and minimal solubility in all other solvents, low salt effects, low buffer concentrations, low-temperature effects, well-behaved cation interactions, higher ionic strengths, and chemical stability [53]. The diluent composition is one of the essential elements impacting sperm quality and keeping sperm fertility for the longest time [54, 55]. Early diluents for ram sperm included citrates, egg yolk, and a monosaccharide such as glucose or fructose and milk [10, 55]. A variety of freezing diluents based on disaccharides (lactose, trehalose), trisaccharides (raffinose), complex polysaccharides (gum Arabic), or other complex compounds (polyvinylpyrrolidone), as well as egg yolk and glycerol, were used to dilute and freeze the ram semen [10, 56]. Among the diluent combinations, tris-based or milk-based diluents are recommended for routine use in rams' semen [8, 10, 11, 57]. To avoid the risk of microbiological contamination, many researchers use soybean lecithin instead of egg yolk in extenders [52, 58, 59]. According to Aboagla and Terada [60], the addition of trehalose or raffinose to the semen extenders plays an important role during the freezing stage of rams' semen cryopreservation. The addition of trehalose or raffinose increases its cryoprotective activity. Bioxcell® (soybean lecithin-based) and AndroMed® were among the commercial diluents used to freeze the ram semen (Tris-based). These are Tris-based and contain phospholipids, citric acid, carbohydrates, antioxidants, and glycerol [13, 58]. However, various homemade diluents have been used to freeze ram sperm [8, 9, 11, 55, 56].

## 6. Cryoprotectant

The diluent used for cryopreservation should contain a cryoprotective preventing agent to protect the spermatozoa from cryogenic injuries during cooling and freezing [10]. Cryoprotectants protect frozen sperm cells by suppressing excessive salt concentrations, limiting cell shrinkage at a given temperature, minimizing intracellular ice formation, and reducing the fraction of the solution frozen at a given temperature [41, 61]. Penetrating (intracellular cryoprotectants) and non-penetrating (extracellular cryoprotectants) cryoprotective agents are the two types of cryoprotective agents. The distinction is due to their capacity to enter sperm cells [52, 62, 63]. Low molecular weight penetrating cryoprotectants (glycerol, dimethyl sulfoxide, ethylene glycol, and propylene glycol) induce membrane lipid and protein reorganization due to increased membrane fluidity, increased dehydration at lower temperatures, and lowered intracellular ice formation, leading to a higher sperm survival rate to cryopreservation [41]. Nonpenetrating cryoprotectants (egg yolk, nonfat skimmed milk, trehalose, amino acids, dextrans, lactose, soybeans, and sucrose) get a high molecular weight, need not pass through the plasma membrane, and only function extracellularly; hence, they can affect cell dehydration. When ram sperm is frozen, glycerol is usually utilized as a cryoprotectant [8–11, 42]. It binds water and lowers the solution's freezing point, reducing ice formation at any temperature [19]. Due to an osmotic stimulation and cell dehydration mechanism, glycerol has an extracellular effect, reducing the volume of intracellular water available for freezing and increasing the survival rate of cryopreserved cells [41]. Based on the concentration and temperature at which it is applied, glycerol is biologically harmful to spermatozoa and toxic to membrane integrity [59]. The toxicity of glycerol limits its use in diluents [19, 41, 52]. Glycerol in high concentrations is hazardous to sperm cells because it can cause osmotic damage. The concentration that provides the best post-thaw survival rate is between 4% and 7%. Because glycerol more easily enters the ram sperm, concentrations larger than 6% are harmful to sperm survival [10]. The amount of glycerol added to the diluents is determined by the extender composition, glycerol addition method, and cooling and freezing rate [2, 10, 41]. According to several studies, adding 3% to 7% glycerol to diluents comprising 5 to 20% egg yolk results in remarkable post-thaw motility restoration (44 to 85%) of ram sperm [12, 14, 64]. Soltanpour and Moghaddam [65] found that diluents having 7% glycerol and 20% egg yolk provided better sperm protection than extenders with 5% glycerol and 5% egg yolk. Higher egg yolk concentrations are used in the semen extender, which may lower glycerol levels. Nur et al. [7] examined the impact of glycerol (6%), propanediol (6%), sucrose (62.5 mM), and trehalose (62.5 mM) in a Tris-based extender with 20% egg yolk on post-thaw sperm quality in ram semen and noticed that all cryoprotectants had such a detrimental effect on sperm motility, morphology, and DNA integrity. Silva et al. [66] compared ram semen made by diluting in a Tris-egg yolk extender containing glycerol (5%), ethylene glycol (3%), or acetamide (3%) and did find that ethylene glycol, like glycerol, was capable of protecting progressive sperm motility, acrosome integrity, and oxidative stress during the freezing process. The usage of 5% glycerol, on the other hand, provided the best protection for plasma membrane integrity. Moustacas et al. [67] mentioned that the extender with 5% glycerol maintained plasma and acrosomal membrane integrity higher when assessing the efficiency of dimethylformamide alone or in combination with glycerol, a cryoprotectant for freezing ram semen. Moreover, when extenders including pure dimethylformamide or more than 2% in combination with glycerol were applied, sperm motilities were reduced to near zero. Egg yolk prevents sperm from cold shock, keeps them motile, prevents acrosomal enzyme loss, and keeps their mitochondrial membranes intact [10, 41]. Egg yolk is often used in freezing semen diluents because of its properties, such as phospholipid concentration, high molecular weight, and low-density lipoprotein fraction. During cryopreservation, the lipid component of egg yolk protects the sperm plasma membrane and acrosome from thermal damage [10, 42]. By forming a spermatozoa-lipoprotein complex, the egg yolk's phospholipids and low-density lipoprotein (LDL) reduce sperm cold shock and hence the cooling injuries to the sperm [42]. When the egg yolk is utilized in conjunction with glycerol in the extender, the protection against cold shock may be increased [52]. Substantially higher egg yolk concentrations do not always imply improved sperm motility preservation. According to Gil et al. [13], a higher egg yolk concentration (over 5%) in a milk-based extender did not improve post-thaw motility. Although the impact depends on the extender composition, egg yolk concentrations ranging from 3–6% (as low as) to 15–20% (as high as) have been used to freeze ram semen [10, 60]. After AI, 15% egg yolk extended frozen-thawed semen increased fertility in sheep [55]. The use of egg yolk and skim milk and their performance after freezing and thawing have been shown to harm sperm quality. When compared to yolk citrates, it is believed that overall, milk used in ram semen preservation has a higher post-thaw survival rate. However, when the outcomes of several freezing procedures were compared, yolk citrates came out on top [68]. Alcay et al. [69] particularly compared lyophilized egg yolk to fresh egg yolk for the freezing of ram semen and reported that lyophilized egg yolk provided similar cryoprotective consequences as fresh egg yolk extender (see Table 1).

## 7. Semen Dilution and Cooling

During the freezing of semen, the selected semen samples are extended with the diluent and involve cooling gradually to 5°C. Dilution and cooling aim to prolong the lifespan and slow down the metabolic activity of the sperm. Semen dilution is done in specified ratios with the proper diluent to ensure that the volume of semen used for insemination has enough sperm per dosage to ensure high fertility without losing cells [119]. Due to an increase in the potassium content of the sperm cell, a too sperm concentrated sperm reduces sperm metabolic activity. When preparing semen for AI, the number of sperms per AI dose should be standardized and diluted using an extender. The cooling process should be done gradually and slowly, which is enough to save the sperm cells. Fast cooling between 30 and 0°C causes sperm damage, the so-called “cold shock” [19]. To prevent sperm cells from cold shock, the initial dilution of semen is done slowly down to 5°C over a few hours (1.5–2.0 h) by gradually adding the diluents, mixing them, and keeping the mixture close to body temperature [57, 120]. Before exposing cells to hyperosmotic conditions caused by dehydration, the cooling rate must be slow enough to allow sufficient cellular dehydration while still fast enough to freeze the remaining intracellular fluid [19, 42]. On the other hand, slow cooling allows water to escape the cells via osmosis, preventing the creation of fatal intracellular ice [121]. If cooled slower, ram sperms in the mid-piece and tail are most sensitive [79]. The optimal cooling rate (from body temperature to 5°C) was −10°C/h, and employing either egg yolk or milk as protective agents resulted in the least cold shock [42]. Semen extenders made with glycerol may be added initially or subsequently in a separate fraction during the dilution and cooling process. In the one-step method, the entire extender is added in the first condition after collecting semen. In the second case, a portion of the extender (without glycerol) is applied after semen collection, and the remainder (with glycerol) is added after cooling before semen freezing (two-step procedure) [30, 122]. Some authors obtained good results when the glycerol fraction was added at 4°C, but in other studies, better results were obtained after glycerol addition at 30–37°C [96, 123]. Although the difference is minor, adding glycerol at 5°C results in improved sperm survival than adding it at 30°C. The sperm stress is decreased to tolerable levels in the stepwise dilution procedure. The addition of cryoprotectants significantly enhances the fraction of surviving sperm compared to the single-step addition of cryoprotectants [19]. This could be owing to glycerol's ability to permeate through the membrane at 30 degrees Celsius. Glycerol is less permeable to cell membranes at 4°C, making it less hazardous. Glycerol's use is restricted due to its toxicity, as previously noted. Equilibration durations with glycerol should be balanced to take advantage of glycerol's cryoprotective qualities while avoiding unnecessary sperm loss before cryopreservation [124]. The appropriate glycerol equilibration time has long been a point of contention among cryobiology professionals. Several studies have found that allowing sperm to equilibrate for several hours using glycerol increased post-thaw motility and fertility. Some writers, however, recommended a glycerol equilibration duration of 1.5–2.0 hours [57, 120].

## 8. Equilibration Time

The total time spermatozoa are in touch with glycerol before freezing is equilibration. However, the equilibration process is not limited to glycerol; it also applies to the other osmotically active extender ingredients. As a result, the equilibration approach can interact with the type of extender (buffer and cryoprotectant) utilized and other cryogenic processes [125, 126]. Different equilibration times ranging from 1 to 5 hours were employed in rams, with varying post-thaw seminal quality [9, 70, 71]. Sharma and Sood [127] and Ranjan et al. [128] found that a 4-hour equilibration period improved post-thaw semen quality. On the other hand, Baruah et al. [129] found no significant variations in sperm motility or acrosomal integrity in semen samples that had been equilibrated for 0.5, 1, or 1.5 hours.

## 9. Semen Freezing

The purpose of freezing semen is to gradually lower the temperature from 5 to −196°C to avoid injuring the sperm cells. When the temperature drops below 5°C and approaches −10°C, the intracellular water freezes, putting sperm cells at risk of forming ice crystals. Since the freezing rate regulates the extent and rate of cell dehydration, it should be as fast as possible. When sperm cells are rapidly cooled, water is not lost quickly enough to maintain balance, and cells that create intracellular ice during cryopreservation die [130, 131]. Suppose the cooling rate is very slow enough. In that case, the sperm cells will be exposed to high solute concentrations for an extended period, resulting in cell dehydration, volume contraction, and no intracellular freezing. All of these variables have an impact on sperm freezing success. The semen extender will determine the best cooling rate and the packaging utilized. Cells and their surrounding media stay unfrozen and superchilled when cooled to around −5°C. The exterior media freezes between −5 and −10°C, yet the cell contents remain unfrozen and supercooled. Because the supercooled water inside the cells has a more potent chemical potential than water in the partially frozen extracellular solution, water flows out of the cells osmotically, and it freezes outside [130]. A reasonable recommendation is to transfer the semen to liquid nitrogen (LN2) for storage at −15°C/min from +5°C to −100°C [132]. Sperm cells were frozen at a quick pace of 15–60°C/min and were found to have a reasonable survival rate. The effective cooling rate for ram sperm has been estimated to be around 20°C/min or more. Semen can be frozen faster or slower. Semen is cooled fast enough to avoid cooling damage yet slowly enough to allow for cell dryness without the production of intracellular ice. The cell dehydration associated with this slow freezing technique may help sperm cells survive, whereas rapid freezing rates are more likely to result in cellular death. A more stable thermodynamic equilibrium characterizes slow freezing. It employs low cryoprotectants, which are commonly linked to chemical toxicity and osmotic pressure [133]. On the other hand, slow freezing appears to be the most crucial aspect of the sheep preservation procedure [2, 133, 134]. Manual freezing or an automatically programmed biofreezer are both options for freezing semen. The semen straws are placed horizontally on a chilled rack and frozen for 8–10 minutes at 4–6 cm above the level of LN2 in the vapor phase (between −75°C and −125°C) in a manual freezer. After thawing, the initial freezing temperature has a considerable impact on spermatozoa motility and velocity; however, the best motility of spermatozoa may be attained at −125°C [135]. Nonetheless, freezing small-diameter straws caused a rapid drop in temperature, leading to intracellular water crystallization, which might induce fewer cell damage during the drop in temperature than delayed freezing, which produces severe dehydration [136]. The size of the straw should be used to determine the freezing level over the liquid nitrogen, according to Chemineau et al. [137]. 0.25 ml straws should be frozen 16 cm above liquid nitrogen for 2 minutes before being lowered to 4 cm for 3 minutes before being plunged into liquid nitrogen for storage, whereas 0.5 ml straws should be frozen 16 cm above liquid nitrogen for 2 minutes before being plunged into liquid nitrogen for storage. Alternative freeze positions and times have been mentioned, like 4–5 cm above liquid nitrogen for 4–5 min, with satisfactory results [26, 138]. Pontbriand et al. [3] found that temperature variations of 6 to 24°C per minute and 10 to 100°C per minute were bearable, implying that ram spermatozoa can endure a wide range of cooling rates. The temperature dropped at a controlled and programmed rate while utilizing an automatic freezing machine, from 4 to −5°C at 20°C/min, −5 to −110°C at 55°C/min, and −110 to −140°C at 35°C/min [80]. Sperm cells are often frozen at a high rate (15–60°C/min), resulting in the best post-thawing results [80]. Generally, sperm mixed in a freezing extender is slowly cooled at a rate of roughly 0.1°C/min from ambient temperature to 5°C and then frozen at a rate of 10–60°C·min-1 to temperatures as lower as −80°C before being stored in liquid nitrogen [139]. Before plunging into LN2, the ultimate temperature should be reduced to at least −130°C, regardless of whether the freezing is done slowly or quickly, to stop all metabolic processes, including thermally driven chemical changes [42] (see Table 2).

## 10. Semen Thawing

The process of thawing is the reversal of freezing, in which the solid phase transforms into a normal liquid phase [42, 141]. Cryopreservation methods and subsequent thawing have an impact on sperm cell survival. Sperm quality is harmed during freezing and thawing because sperm cells are exposed to two crucial temperature zones (−15 to −60°C): once during cooling to −196° C and again while thawing. In general, roughly 40–50 percent of the sperm population dies after freezing and thawing [19]. Survivability can be lowered from 85.6 to 34.3 percent in buck frozen-thawed sperm. While frozen-thawed ram semen may contain a large proportion of motile cells (40–60%), only around 20–30% of them are physiologically active [10, 19, 42]. The cells are pushed to retract their route across the numerous environments encountered during thawing, and the influx of water that results can induce membrane breakage. The warming phase (thawing) is equally as critical to the spermatozoa's survival as the initial cooling phase during freezing during the freeze-thawing of semen [10]. The combination of processing elements such as glycerol content, freezing rate, and packaging method determines the ideal thawing rate in most cases. It is worth noting if the cooling rate employed to freeze the sperm was high enough to induce intracellular freezing or low enough to cause cell dehydration [142, 143]. If the cooling rate was high, rapid thawing would be required to prevent any intracellular ice in the sperm cell from recrystallizing [136]. When sperms are thawed quickly, they are exposed to a concentrated solute and cryoprotectant for a short period of time, and the restoration of intracellular and extracellular equilibrium is faster than when they are thawed slowly. This topic was highlighted by Hammerstedt et al. [141], who noted that the rate of semen thawing must be adjusted for varied freezing rates in order to get the best sperm cell survival rate. Slow thawing (35°C for 12 sec) leads to 63% and 50% post-thawing sperm motility and membrane integrity, respectively [144]. Fast thawing (70°C for 5 sec) leads to higher post-thawing sperm motility and membrane integrity (67 and 50%, respectively).

Evans and Maxwell [30] discovered that thawing ram sperm at 38–42°C for 15–30 seconds results in sperm motility, acrosome integrity, and sperm cell fertility that are also similar. However, thawing at higher temperatures (60–75°C for 8 seconds) may result in equivalent sperm motility, acrosome integrity, and sperm cell fertility after thawing. Pontbriand et al. [3] showed no difference in spermatozoal motility, progressive status ratings, or acrosomal integrity when thawing straws in a water bath at a higher rate (60°C for 8 sec) versus a slower rate (37°C for 20 sec). When sperms were thawed at 50°C for 9 seconds instead of 70°C for 5 seconds, there was no difference in motility or membrane integrity, showing that thawing at 70°C for 5 seconds was superior to thawing at 37°C for 20 seconds. In farm conditions, thawing at a lower temperature may make it easier to use frozen-thawed ram semen. Several studies have demonstrated that a longer thawing period is preferable to a shorter thawing period [145, 146]. A semen straw is usually thawed by immersing it in a 37°C water bath for 12–30 seconds [132]. At temperatures above 37°C, temperature and time become significantly more hazardous, as these high temperatures might result in massive sperm mortalities if thawing is done wrong [147]. When sperm cells are exposed to temperatures between −5 and 15 degrees Celsius, they are damaged [79]. Individual ejaculates of ram sperm are only suitable for preservation and insemination if the percentage of forward moving spermatozoa is greater than 40% after thawing and 30% after 5–6 hours of incubation [30].

## 11. Conclusions

During the cryo-freezing procedure, ram spermatozoa are more vulnerable to cold shock [2]. To achieve optimum sperm quality, various types of diluents, dilution methods, and cooling-freezing protocols have been endlessly tried by researchers worldwide. In this review, we summarized different cooling rates on ram semen cryopreservation. Personnel skills, quality chemical and cryo-protectants, and proper instrument function impede laboratory production of higher quality frozen ram sperm. The protocol that provides better advantages in ensuring less damage to sperms cells and better post-thaw quality should be adopted and optimized as a lab-specified protocol.

## Figures and Tables

**Table 1:** Effects of cryoprotectants and thawing temperature on Post-thaw sperm motility of ram semen.

Breed	Collection method	Egg yolk	Glycerol (%)	Thawing time and temperature	Post-thaw motility (%)	Reference
Merino ram	AV	6%	5%	37°C for 30 s	44.5 ± 1.9	[70]
					47.9 ± 2.0	
					61.4 ± 1.9	
Bangladeshi rams	AV	20%	7%	7°C for 20 s	41.7 ± 2.9	[71]
			5%		56.3 ± 2.0	
Mehraban rams	AV		7%	37°C for 30 s	52.5% ± 1.8	[72]
Zandi ram	AV		5%	37°C for 30 s	46.20 ± 1.59	[73]
			7%			
	EE	20%	6%	37°C for 30 s	53.00 ± 1.27	[69]
Assaf rams	EE	10%	4%	65°C for 6 s	48.7 ± 20.9	[74]
		20%	8%			
Zandi rams	AV	20%	7%	37°C for 30 s	48.43	[75]
Chal rams	AV	10%	5%	37°C for 20 s	74.6 ± 7.8	[76]
					61.2 ± 11.1	
Hemşin rams	EE	15%	5%		29.8 ± 2.76	[77]
Dorper rams	AV		3%	37°C for 30 s	22.7 ± 4.5	[78]
			7%			
	AV	6%	4%	40°C for 30 s	33 : 3 ± 8 : 02	[79]
Awassi rams		20%	6%	37°C for 30 s	51.2 ± 1.9	[7]
	AV		7%			[80]
Indigenous Bangladeshi rams	AV	10%	7%	39°C for 14 s	62.0 ± 0.6	[8]
Leccese rams	AV	20%	5%	37°C for 30 s	29.4 ± 2.9	[81]
	AV	20%		37–39°C for 20–30 s	29.2	[82]
Pampinta rams	AV	10%	6%	37°C for 10 s		[83]
Bakhtiari rams		20%	7%	37°C for 10 sec	51.8 ± 2.9	[52]
Santa Inês crossed rams		16%	5%	37°C for 30 s		[67]
Awassi rams	EE			37°C for 30 s	42.8 ± 8.8	[84]
Awassi rams	EE	20%	6%	37°C for 30 s	48.0 ± 5.6	[54]
Santa Inês rams	AV	20%	5%	37°C for 30 s		[85]
Sarda rams	AV	20%		39°C for 20 s		[86]
Crossbred rams	AV	20%	7%	37°C for 30 sec	56.94 ± 0.79	[87]
Pampinta ram	AV	10%	3%	37°C for 10 s		[88]
Rahmani rams	AV	20%	6 or 3%	37°C for 30 s	41	[89]
Pampinta rams	AV	10%	6%	37°C for 10 s	58.3 ± 2.4	[90]
Merino rams	AV	15%	5%	37°C for 2 min		[91]
Bakhtiari rams	AV	20%	8%	37°C for 10 s	50.1 ± 2.1	[92]
Suffolk ram	AV	15%	5%	37 C for 20–30 sec		[58]
Merino rams	AV	15%	5%	At 37°C for 20 s	50.9 ± 3.1	[93]
Akkaraman rams	AV	10%	5%	37°C for 20 s	50.00 ± 1.58	[94]
Akkaraman rams	AV	10%			39.5 ± 2.73	[95]
Churra rams	AV	20%	6%	65°C for 6 s	64.1 ± 10.8	[12]
Santa Inês rams	AV	10%	6%	37°C for 30 s	38.1 ± 14.8	[85]
Île-de-France and lacaune rams	AV	20%	4%	37 to 38°C for 30 sec	38	[96]
Leccese dairy breed of rams	AV	20%	4%		71	[97]
Crossbred ram	AV	20%	2.5%, 5%, 10%, 15%, 20%, 25%, 30%, 35%, 40%	39°C for 30 s		[98]
Manchega ram	AV			37°C for 20 s	55.06 ± 1.22	[99]
Bangladeshi ram		10%	7%		41.4 ± 0.7	[100]
Suffolk ram	AV			38.5°C, 30 s	44.2	[101]
Taleshi rams	AV	20%	7%	37°C for 30 s	23.6 ± 0.6	[102]
Norwegian crossbred rams	AV	5%	7%	35°C for 15 s		[103]
Dorper ram	AV	20%	5%	40°C for 20 s	64.4 ± 0.8	[104]
Norwegian crossbred rams		5%	7%	35°C for 12 sec		[105]
Merino rams	AV	10%	4%	20 s at 37°C	65.0 ± 4.2	[106]
Dorset crossbred rams		20%	4%	37°C for 20 s or 60°C for 8 s	47.0 ± 1.2	[3]
Santa Inês rams	AV	20%	5%	37°C for 30 s		[66]
Indigenous rams		20%	5%	37°C for 20 sec		[107]
Portuguese Serra da Estrela and Saloia, rams	AV	15%	5.3%		46.5 ± 5.3	[55]
Suffolk rams	AV	15%	5%		38.8 ± 4.3	[108]
Merino-Sakiz crossbreed rams	EE	15%	5%		40.0 ± 3.87	[109]
		20%	6%	37°C for 30 s		[110]
Akkaraman rams		10%	5%		54.23 ± 0.09	[111]
Merino of Palas rams	AV	20%	5%	37°C for 30 s	56.66 ± 1.66	[112]
Zandi rams	AV	20%	7%	37°C for 30 s	48.43	[75]
Malpura ram	AV	15%	6%	60°C for 6 s	47.96 ± 3.0	[113]
Malpura and Bharat Merino				50°C for 10 s	49.0	[114]
					53.3	
					58.4	
Garole ram		15%	6%	50°C for 10 seconds	70.4 ± 2.29	[115]
Suffolk rams		15%	6%		46.7	[116]
Dorset rams		15%	5%	37°C for 30 s	33.2	[117]
Moghani sheep		5%	5%	37°C for 30 s		[118]

EE = electroejaculation; AV = artificial vagina.

**Table 2:** Comparison of various protocols for freezing of ram semen.

Freezing step	Diluents	Equilibration time	Freezing rate	Concentration/ dilution	Reference
Three	Tris/tes/glucose (TTG) solution	5°C for 3 h	40°C/min (+5°C to −35°C)	100 × 10^6^ sperm/mL	[70]
			17°C/min (−35°C to −65°C)		
			3°C/min (−65°C to −85°C) and finally −196°C		
Three	Tris/tes/glucose (TTG) solution	5°C for 3 h	4°C/min (+5°C to −5°C)	100 × 10^6^ sperm/mL	[70]
			25°C/min (−5°C to −110°C)		
			35°C/min (−110°C to −140°C) and finally −196°C		
Two	Tris/tes/glucose (TTG) solution	5°C for 3 h	5°C/min (+5°C to −10°C)	100 × 10^6^ sperm/mL	[70]
			60°C/min (−10°C to −130°C) and finally −196°C		
One	Tris-citrate-fructose-egg yolk and Triladyl®	5°C for 4 h	−15.26°C/min (+5°C to −140°C) and finally −196°C	800 × 10^6^ sperm/ml	[71]
Two	Tris-citrate-fructose-egg yolk and Triladyl®	5°C for 4 h	−30°C/min (+5°C to −80°C)	800 × 10^6^ sperm/ml	[71]
			−11.33°C/min (−80°C to −140°C) and finally −196°C		
Three	Tris-citrate-fructose-egg yolk and Triladyl®	5°C for 4 h	−11.33°C/min (+5°C to −80°C)	800 × 10^6^ sperm/ml	[71]
			−26.66°C/min (−80°C to −120°C)		
			−13.33°C/min (−120°C to −140°C) & finally −196°C		
One	Tris-citrate-fructose media	4°C for 2 h	Vapor freezing 5 cm above the LN2 for 12 min, then immersed directly into liquid nitrogen at − 196°C	4 × 10^8^ sperm/ml	[72]
Two	Tris-citrate-fructose media	4°C for 2 h	3°C/min (+5°C to −8°C)	4 × 10^8^ sperm/ml	[73]
			15°C/min (−8°C to −120°C) & finally −196°C		
Two	Tris-citrate-fructose-egg yolk	5°C for 120 min	3°C/min (+5 to −8°C)	1 : 2 (semen/extender)	[69]
			15°C/min (−8 to −120°C) & finally −196°C		
One	Tris-fructose-egg yolk media	5°C for 2 h	−20 ◦C/min (5°C to −100°C) & finally -196 °C	100 × 10^6^ sperm/ml	[74]
One	Tris-citrate-fructose media	4°C for 2 h	Vapor freezing 5 cm above the LN2 for 12 min, then immersed directly into liquid nitrogen at −196°C	350 × 10^6^ sperm/ml	[75]
Two	Tris-citrate-fructose-egg yolk	5°C for 80 min	−0.3°C/min (5°C to −10°C)	4 × 10^8^ sperm/ml	[76]
			−25°C/min (−10°C to −150°C) & finally −196°C		
One	Tris-citrate-fructose-egg yolk	5°C for 3 h	Vapor freezing 4 cm above the LN2 for 15 min, then immersed directly into liquid nitrogen at −196°C	4 × 10^8^ sperm/ml	[76]
One	Tris-citrate-fructose-egg yolk		Vapor freezing 4 cm above the LN2 for 10 min, then immersed directly into liquid nitrogen at −196°C	80 × 10^6^ sperm/ml	[77]
One	Steridyl® (Minitube, Germany)	5°C for 2 h	Vapor freezing 7 cm above the LN2 for 15 min, then immersed directly into liquid nitrogen at −196°C	100 × 10^6^ sperm/ml	[78]
Two	Hepes–glucose buffer		−5°C/min (+5°C to −5°C)	20 × 10^6^ sperm/ml	[79]
			−50°C/min (−5°C to −50°C) & finally −196°C		
Two	TRIS-based extender	5°C for 4 h	3°C/min (+5°C to −8°C)	1 : 1 (semen/extender)	[7]
			25°C/min (−8°C to −120°C) & finally −196°C		
Two	Skim milk and egg yolk	5°C for 90 min	−5°C/min (5°C to −25°C)	800 × 10^6^ sperm/ml	[80]
			−50°C/min (−25°C to −130°C) & finally −196°C		
One	Tris, fructose, egg yolk or Triladyl®	4°C for 4 h	Vapor freezing 4 cm above the LN2 for 6 min, then immersed directly into liquid nitrogen at −196°C	400 × 10^6^ sperm/mL	[9]
One	Tris or milk-based diluent		Vapor freezing at −75°C for 7 min, then immersed directly into liquid nitrogen at −196°C	50, 100, 200, 400, 500, or 800 × 10^6^ sperm/mL	[97]
One	TEST buffer	5°C for 4 h	Vapor freezing 5–7 cm above the LN2 for 10 min, then immersed directly into liquid nitrogen at −196°C	1 : 4 (semen/extender)	[82]
One	Tris-citrate modified solution	5°C for 2 h	Vapor freezing at −100°C, then immersed directly into liquid nitrogen at −196°C	1 × 10^9^ sperm/mL	[83]
One	Tris-citrate-fructose-egg yolk	4°C for 2 to 3 h	Liquid nitrogen vapor for 12 min, then immersed directly into liquid nitrogen at −196°C	1 × 10^9^ sperm/mL	[52]
One	Tris-citrate-glucose-egg yolk-glycerol media	5°C for 3 h	Vapor freezing 3 cm above the LN2 for 15 min, then immersed directly into liquid nitrogen at −196°C	100 × 10^6^ sperm/mL	[67]
Two	Bioxel	5°C for 3 h	5°C/min (5°C to −20°C)	1 : 5 (semen/extender)	[84]
			25°C/min (−20 to −120°C) & finally −196°C		
One	TRIS-egg yolk	5°C for 4 h	Vapor freezing at −110°C for 10 min, then immersed directly into liquid nitrogen at −196°C	1 : 1 (semen/extender)	[54]
One	Tris-egg yolk-glycerol	5°C for 3 h	−15°C/min (+5°C to −120°C) finally −196°C	240 × 10^6^ sperm/mL	[140]
One	Tris-citrate-fructose-egg yolk	4°C for 140 min	Freezing on dry ice, then immersed directly into liquid nitrogen at −196°C	400 × 10^6^ sperm/ml	[86]
Three	Tris-citrate-fructose-egg yolk	4–5°C for 4 h	−5°C/min (+4°C to −10°C)	150 × 10^6^ per straw	[87]
			−40°C/min (−10°C to −100°C)		
			−20°C/min (−100°C to −140°C) & finally −196°C		
One	Tris-citrate-fructose-egg yolk- glycerol media		Vapor freezing at −100°C, then immersed directly into liquid nitrogen at − 196 °C	1 × 10^9^ cells/ml	[88]
One	Tris-citrate-fructose-egg yolk-glycerol media	5°C for 4 h	Vapor freezing 5 cm above the LN2 for 10 min, then immersed directly into liquid nitrogen at −196°C	200 × 10^6^ sperm/ml	[89]
One	Tris-citrate-fructose-egg yolk	5°C for 2 h	Vapor freezing, then immersed directly into liquid nitrogen at −196°C	4 x 10^8^ sperm/ml	[90]
One	Tris-citrate-glucose-egg yolk-glycerol media		Frozen in pellets on dry ice (−79°C), then immersed directly into liquid nitrogen at −196°C	400 × 10^6^ sperm/ml	[91]
One			Vapor freezing 5 cm above the LN2, then immersed directly into liquid nitrogen at −196°C	100–200 × 10^6^ spz/ml	[92]
One	Tris-citrate-fructose-egg-yolk-glycerol media and AndroMed	4°C for 2–3 h	Vapor freezing (–125°C to –130°C) for 3–4 min, then immersed directly into liquid nitrogen at −196°C	250 × 10^6^ per ml	[58]
One	Tris-citrate-fructose-egg yolk-glycerol media	5°C for 3 h	Vapor freezing 4 cm above the LN2 for 15 min, then immersed directly into liquid nitrogen at −196°C	4 × 10^8^ sperm/ml	[93]
One	Tris-citrate-fructose-egg yolk-glycerol media	5°C for 2 h	Vapor freezing 4.5 cm above the LN2 for 15 min, then immersed directly into liquid nitrogen at −196°C	4 × 10^8^ sperm/ml	[94]
One	Tris-citrate-fructose-egg yolk-media		Vapor freezing (−100°C to −120°C), then immersed directly into liquid nitrogen at −196°C	4 × 10^8^ sperm/ml	[95]
One	Tris-citrate extender		Vapor freezing 5 cm above the LN2 for 10 min, then immersed directly into liquid nitrogen at −196°C	100 × 10^6^ sperm/ml	[12]
One	Tris-egg yolk extender	5°C for 90 min	12.5°C/min (5 to −120°C)	100 × 10^6^ sperm/mL	[85]
Two		5°C for 4 h	20°C/min to −100°C	200 × 10^6^ sperm/ml	[99]
			10°C/min (−100°C to −140°C)		
One	Tris-fructose-citrate-egg yolk	4°C for 4 h	Vapor freezing 5–6 cm above the surface of the liquid nitrogen for 5–6 (temperature −80°C) minutes, then immersed directly into liquid nitrogen at −196°C		[100]
Four	BullXcell® AndroMed®	2 h at 4°C	Vapor freezing (1st phase, distance 15 cm, 4 min; 2 nd phase, distance 9.5 cm, 5 min; 3rd phase, distance 5 cm, 6 min; 4th phase, distance 1.5 cm, 8 min)		[101]
One	Tris-citrate-glucose-egg yolk- glycerol media	5°C for 2 h	Vapor freezing 4.5 cm above the LN2 for 13 min, then immersed directly into liquid nitrogen at −196°C	600 × 10^6^ sperm/ml	[102]
Two	Milk-based extender	60–90 min at 5°C	5°C/min (5°C to −10°C)	400 × 10^6^ sperm/ml	[103]
			60°C/ min (−10°C to −130°C) & finally −196°C		
One	Tris-citrate-egg yolk-glycerol media	5°C for 120 min	20°C/min (5 to −120°C)	100 × 10^6^ sperm/mL	[104]
Two	Milk-based extender	5°C for 90 to 120 minutes	5°C/min (5°C to −10°C)	1000 × 10^6^ sperm/mL	[105]
			60°C/min (−10°C to −130°C) & finally −196°C		
One	Tris-citrate-glucose-egg yolk media	5°C for 90 min	Vapor freezing −100°C for 10 min, then immersed directly into liquid nitrogen at −196°C	1 x 10^9^ sperm/mL	[106]
One	Tris-based extender		20°C/min (4°C to −100°C) finally −196°C	1 : 3 or 1 : 6 (semen/extender)	[3]
One	Tris-based extender		Vapor freezing 5 cm above the LN2 level (−150°C) for 20 min., then immersed directly into liquid nitrogen at −196°C	1 : 3 or 1 : 6 (semen/extender)	[3]
One	Tris-egg yolk-glycerol	5°C for 3 h	Vapor freezing from 5 to −120°C in 15 min, then immersed directly into liquid nitrogen at −196°C	400 × 10^6^ sperm/mL	[66]
Three	Tris-citrate-fructose egg yolk media	5°C for 4 h	11.33°C/min (+5°C to −80°C)	400 or 800 × 10^6^ sperm/mL	[107]
			26.66°C/min (−80°C to −120°C)		
			13.33°C/min (−120°C to −140°C) & finally −196°C		
One	Tris-citrate-fructose egg yolk media	4°C for 2-3 h	Vapor freezing (−125 to −130°C) for 3-4 min, then immersed directly into liquid nitrogen at −196°C		[108]
One	Tris-based extender	5°C for 2.5 h	Vapor freezing 5 cm above the LN2 for 15 min, then immersed directly into liquid nitrogen at −196°C	800 × 10^6^ sperm/ml	[109]
One	Tris-citrate—fructose-egg yolk-glycerol media	4°C for 4-5 h	Vapor freezing for 10 min, then immersed directly into liquid nitrogen at −196°C	400 × 10^6^ sperm/ml	[110]
One	Tris-citrate—glucose-egg yolk-glycerol media	5°C for 2.5 h	Vapor freezing 12 cm and 4 cm above liquid nitrogen, then immersed directly into liquid nitrogen at −196 °C	4 × 10^8^ sperm/ml	[112]
One	Soybean lecithin-based semen extender	4°C for 2 h	Vapor freezing 5 cm above the LN2 for 12 min, then immersed directly into liquid nitrogen at −196°C	350 × 10^6^ sperm/ml	[75]
One	Tris—fructose-egg yolk-glycerol media	5°C for 3, 10 and 22 h	−25°C/min (5 to −125°C) finally −196°C	800 × 10^6^ sperm/ml	[113]
One	TEST-yolk-glycerol extender	5°C for 2 h	−25°C/min (5 to −25°C) finally −196°C	1000 × 10^6^ sperm/ml	[114]
One	TEST-yolk-glycerol extender	5°C for 2 h	−25°C/min (5 to −75°C) finally −196°C	1000 × 10^6^ sperm/ml	[135]
One	TEST-yolk-glycerol extender	5°C for 2 h	−25°C/min (5 to −125°C) finally −196°C	1000 × 10^6^ sperm/ml	[114]
One	Article	5°C for 2 h	25°C/min (5°C to −125°C) finally −196°C	1 x 109/ml sperms/ml	[115]
One	Tris—citrate-fructose-egg yolk-media	5°C for 2 h	30°C/min (5°C to −150°C) finally −196°C	400 × 10^6^ sperm/ml	[117]
One	Tris–citric acid–fructose–yolk extender	5°C for 3.5 h	Vapor freezing 4–5 cm above the LN2 for 10 min, then immersed directly into liquid nitrogen at −196°C	1 × 10^9^ sperm/ml	[118]
